# Inferior vena cava prosthetic replacement in a patient with horseshoe kidney and metastatic testicular tumor: technical considerations and review of the literature

**DOI:** 10.1186/1471-2490-14-40

**Published:** 2014-05-22

**Authors:** Pietro Rispoli, Paolo Destefanis, Paolo Garneri, Gianfranco Varetto, Beatrice Lillaz, Claudio Castagno, Patrizia Lista, Libero Ciuffreda, Dario Fontana

**Affiliations:** 1 Division of Vascular Surgery, Department of Surgical Sciences, Città della Salute e della Scienza– Molinette Hospital, University of Turin, Turin, Italy; 2 Division of Urology, Department of Surgical Sciences, Città della Salute e della Scienza– Molinette Hospital, University of Turin, Turin, Italy; 3 Division of Oncology, Department of Oncology and Hematology, Città della Salute e della Scienza –Molinette Hospital, Turin, Italy; 4 Divisione Universitaria di Chirurgia Vascolare, Molinette Hospital, C.so Bramante, 88/90-10126 Torino, Italy

**Keywords:** Testis, Neoplasm GCT, PTFE

## Abstract

**Background:**

Seminomatous and non-seminomatous Germ Cell Tumors (GCT) of the testis are a rare cancer, with an estimated incidence of 56.3 per million white males and 10 per million black males in the United States.

The association between non-seminomatous GCT and horseshoe kidney is a rare event and is seen in about 1.3% of patients requiring retroperitoneal lymph node dissection. To our knowledge, no cases have been reported in which replacement of the IVC was also necessary.

**Case presentation:**

We report the case of a 22-year-old man with horseshoe kidney and metastatic non-seminomatous germ cell tumor involving the wall of the inferior vena cava.

Following post-chemotherapy retroperitonal lymph node dissection, the inferior vena cava was replaced with an expanded polytetrafluoroethylene graft.

At 2-years follow-up, the patient was in good health and the graft was patent. No clinical or diagnostic signs of renal impairment or recurrence of neoplastic disease were noted.

**Conclusion:**

Radical surgery is warranted in patients with non-seminomatous germ cell tumor metastasizing to the retroperitoneal lymph nodes. When vena cava replacement is required, and the situation is further complicated by horseshoe kidney, as in this case, surgical technique will rely on multidisciplinary surgical treatment planning by a team composed of urologists, vascular surgeons and oncologists.

## Background

Seminomatous and non-seminomatous Germ Cell Tumors (GCT) of the testis are a rare cancer, with an estimated incidence of 56.3 per million white males and 10 per million black males in the United States. The annual incidence of seminomatous GCT is about 32 cases per million and that of non-seminomatous GCT about 27 cases per million [1]. The American Cancer Society estimates 8,820 new cases of testicular cancer will be diagnosed in the United States in 2014 (http://www.cancer.org/cancer/testicularcancer/detailedguide/testicular-cancer-key-statistics).

Testicular cancer is the most frequent type of testicular cancer in males between 20 and 35 years of age; the 5-year survival rate of seminomatous GCT is 72-80% and that of non-seminomatous GCT is 48-92% depending on prognostic class [2]. The factors that have contributed most to improving survival are accurate tumor staging at diagnosis and appropriate early treatment combining chemotherapy, radiotherapy (in seminomatous GCT), surgery, and careful follow-up. With an aggressive multimodality approach combining the use of cisplatin chemotherapy and surgery, survival rates have improved to 65-85% in patients with poor prognosis, depending on initial extension of disease [3,4].

Surgery with either post-chemotherapy lymph node dissection or residual tumor resection has become a mainstay in the treatment of non-seminomatous GCT presenting one or more residual masses after chemotherapy. As post-chemotherapy surgery poses particular challenges and often requires ad hoc vascular intervention, e.g., vena cava or aortic graft replacement, patients should be referred to a specialized surgery center with expertise in hepatic resection, vessel replacement, spinal neurosurgery, and thoracic surgery. The benefit to patients treated at such interdisciplinary centers is a significant reduction in perioperative mortality from 6 to 0.8% [5] and local recurrence from 16 to 3% and an overall higher rate of complete resection when treated by a urologic surgeon [6].

The concurrent presentation of non-seminomatous GCT with retroperitoneal metastasis involving the inferior vena cava and horseshoe kidney, a congenital disorder, is a rare event that further complicates surgical treatment of the tumor. To our knowledge, this is the first such case to be reported.

## Case presentation

**Figure 1:**
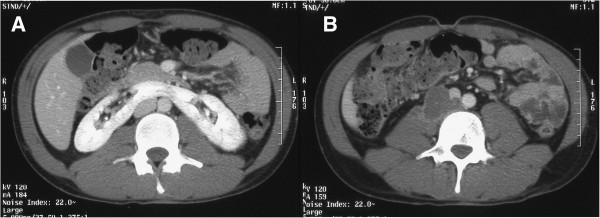
**Retroperitoneal right laterocaval mass.** CT shows the coexistence of a horseshoe kidney **(A)** and a retroperitoneal right laterocaval mass of the maximum diameter of 7 × 9 cm, indissociable from the iliopsoas muscle and without clear signs of infiltration of the caval wall **(B)**.

A 22-year-old man underwent right radical orchiectomy for a testicular mass; the histopathological diagnosis was pure teratoma of the testis. Computed Tomography (CT) with contrast material of the abdomen, chest and head for tumor staging showed metastases to the liver, lungs, retroperitoneal lymph nodes, and brain. The imaging studies also revealed a right laterocaval retroperitoneal mass (largest diameter 7 × 9 cm) invading the iliopsoas muscle but without clear signs of caval wall infiltration (Figure 1). An incidental imaging discovery was a horseshoe kidney with a parenchymatous isthmus. The level of Beta-Human Chorionic Gonadotropin (BHCG) was 2,250,000 IU/L (normal values < 5 IU/L in males) and that of alpha-fetoprotein 2 ng/ml (normal values < 10 ng/ml in adult males).

The patient subsequently underwent three cycles of chemotherapy with etoposide and cisplatin; a fourth cycle of EP was suspended due to the occurrence of a bacterial endocarditis. After the improvement of clinical conditions, the patient underwent a salvage chemotherapy with four cycles of etoposide, ifosfamide and cisplatin, during which the BHCG level decreased to 86 IU/L. On Positron-Emission Tomography (PET), elevated metabolic activity was absent in the lung and liver lesions but present in the laterocaval retroperitoneal lymph node mass.

On AngioCT prior to surgery for removal of the residual retroperitoneal mass, the mass (largest diameter 5-6 cm) was found to be continuous with the inferior vena cava and extend to the inter-aorto-caval area. The images also showed, besides the known horseshoe kidney, two right renal arteries, one left renal artery, and one renal vein on each side draining into the inferior vena cava.

**Figure 2:**
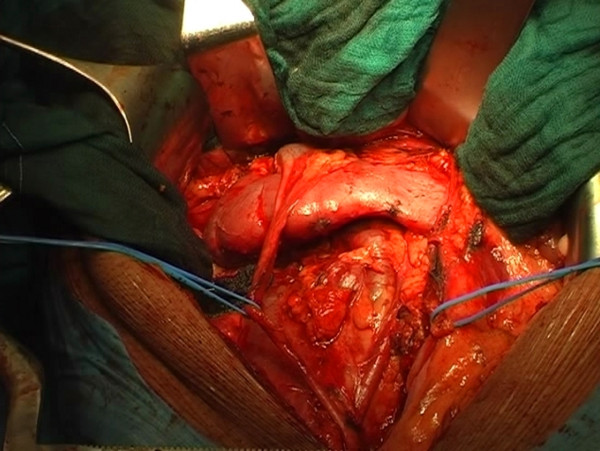
**Horseshoe kidney and cava vein.** The retroperitoneal mass, of diameter of about 6 cm, was infiltrating the psoas muscle and also the lateral wall of the cava vein that was interested in full-thickness to about 5 cm of extension, from its portion below the isthmus renal up to 3-4 cm from the ileo-caval confluence.

The retroperitoneal mass was removed via transperitoneal surgical access. After a puboxiphoid incision and V opening of the retroperitoneal cavity, the right side of the horseshoe kidney and the two right renal arteries were isolated. The retroperitoneal lymphatic mass (largest diameter about 6 cm) was found to infiltrate the psoas muscle and the lateral wall of the inferior vena cava along its entire thickness for about 5 cm, from the segment underneath the renal isthmus till 3-4 cm from the iliocaval confluence (Figure 2).

**Figure 3:**
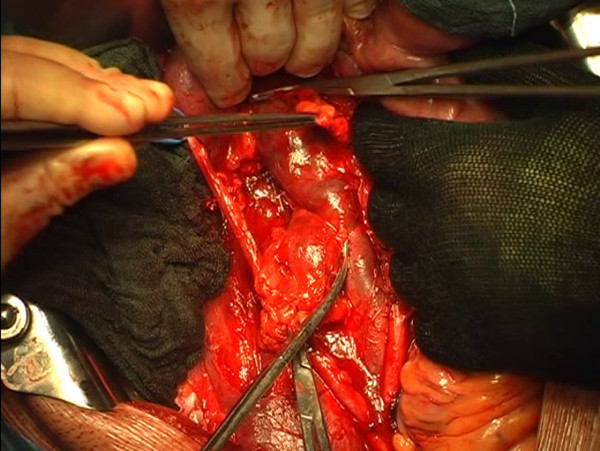
**The caval lymph-node dissection begins.** The right side of the horseshoe kidney was dislodged from the posterior planes of the abdominal cavity, flipped superomedially en bloc with its vascular structures to expose the mass.

**Figure 4:**
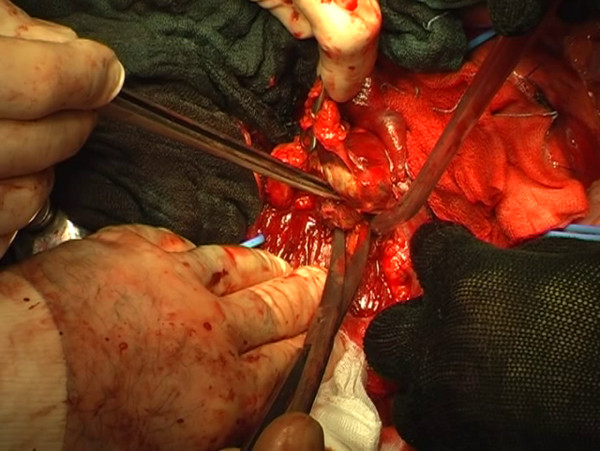
The caval mass is dissected, but it infiltrates the psoas muscle that is partially sectioned with the mass.

**Figure 5:**
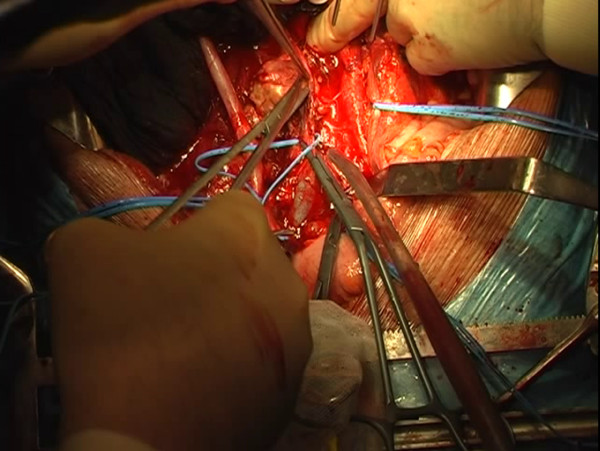
During the dissection, an attempt to free the cava vein from the mass is made, but the caval wall is massively infiltrated by the lesion.

**Figure 6:**
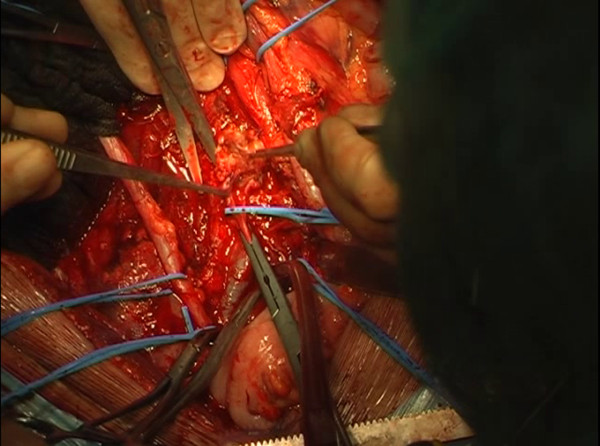
The segment of inferior vena cava infiltrated by the mass is then removed.

**Figure 7:**
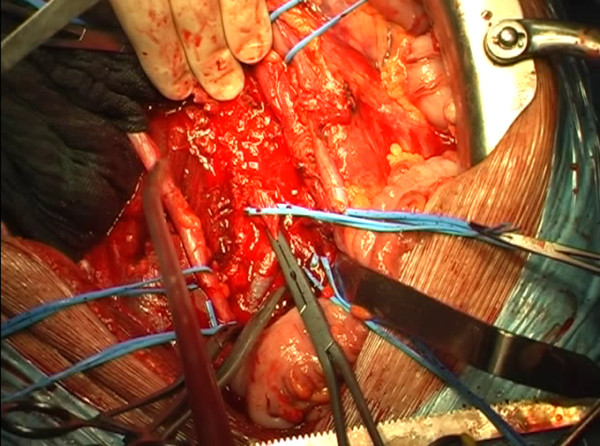
The operative field after the complete removal of the residual mass, the inferior vena cava and a part of psoas muscle.

**Figure 8:**
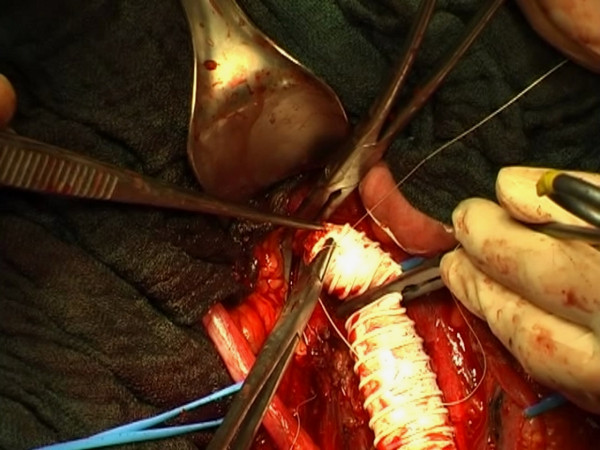
**Cava prosthetic replacement.** Removal of the mass infiltrating the wall of the vena cava and inferior vena cava replacement for a section of about 10 cm with a 16 mm ringed PTFE prosthesis.

The right external iliac lymph nodes, laterocaval, interaortocaval and presacral lymph nodes were removed. The right side of the horseshoe kidney was dislodged from the posterior planes of the abdominal cavity, flipped superomedially en bloc with its vascular structures to expose the mass (Figure 3). The lymph node mass infiltrating the caval wall was removed (Figures 4, 5, 6, 7) and an approximately 10 cm long section of the inferior vena cava was replaced with a ringed PTFE (PolyTetraFluoroEthylene) graft (16 mm) (Figure 8). Anticoagulant therapy with low-molecular weight heparin at the therapeutic dose was initiated. Final pathology showed tumor necrosis comprehending a small area of chondroid vital tissue for the mass infiltrating inferior vena cava and psoas muscle. All other resected lymph-nodes resulted negative.

**Figure 9:**
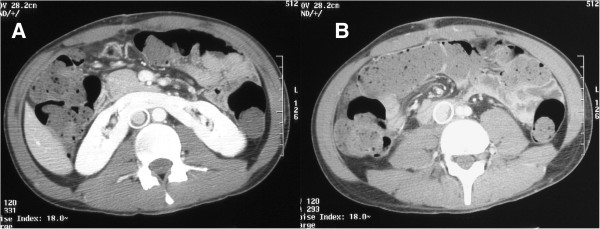
**Follow up imaging.** The control CT at 1 year **(A and B)** demonstrates the absence of the lesion and the patency of the prosthesis.

Control CT at 1 month postoperative showed graft and inferior vena cava patency, a reduction of about 20% in the volume of the lung lesions as compared to the CT scan obtained before chemotherapy, and no recurrence of neoplastic abdominal tissue. The graft patency and absence of abdominal neoplastic tissue was confirmed at 1 year AngioCT (Figure 9).

The patient then underwent removal of the brain metastases secondary to GCT with choriocarcinomatous features, followed by one cycle of chemotherapy with paclitaxel, ifosfamide, and cisplatin. At 18 months the patient underwent resection of bilateral lung lesions. At 2 years follow-up, the patients was in good health and virtually disease free (normal value of tumor markers). Control AngioCT confirmed transformation of the liver lesions into cysts. The inferior vena cava and the graft were both patent, and no signs of renal insufficiency were noted. The patient is still under oral anticoagulant therapy. Renal function was unchanged as compared to pretreatment status.

### Discussion

The International Germ Cell Cancer Collaborative Group (IGCCCG) classifies GCT as a tumor having good, medium, or poor prognosis on the basis of variables including primary tumor site, presence or absence of metastases, levels of AFP (Alpha-FetoProtein), BHCG, and LDH [2]. In view of these variables, the GCT in our patient was classified as carrying a poor prognosis owing to the presence of extrapulmonary lesions and the extremely high level of BHCG (2,250,000 IU/L) at diagnosis. According to the guidelines of the European Association of Urology (March 2011) and the National Comprehension Cancer Network (NCCN 2012), in cases of non-seminomatous GCT treated with orchiectomy and chemotherapy but with residual retroperitoneal lymph nodes >1 cm, removal of the residual mass is warranted and defined as “desperation surgery” in some cases [7,8]. Of fundamental importance is radical surgery [9], with ad hoc vascular surgery procedures for caval and aortic replacement [4].

Recent studies have shown that about 7% of patients who had received post-chemotherapy retroperitoneal lymph node dissection will require resection of the IVC [10] because of tumoral infiltration into the cava. Recently, Winter et al. evaluated 339 cases with an absolute indication of residual tumor resection after chemotherapy in patients with advanced stage GCT. Surgery of the IVC was necessary in 34 patients (about 10%), in 56% of which vena cava resection and graft placement were required. Resection and interruption of the IVC without reconstruction is possible only in cases of previous caval obstruction and the formation of collaterals which provide for sufficient venous drainage. No complications, e.g., lower limb oedema, decreased renal function, developed in any of the patients undergoing graft reconstruction of the IVC [11]. However, Gloviczki et al. reported a 54% patency rate at 2 years after iliacocaval reconstruction with PTFE grafts [12].

An alternative to grafting may be primary ligation of the IVC without replacement of the removed segment; however, this is associated with a higher post-operative complications rate, a mortality rate of 9%, and severe lower limbs oedema in 36% of cases. Symptoms secondary to chronic venous insufficiency, including secondary varicose veins of the lower limbs, scrotum and abdomen, have been reported in up to 50% of cases. A recent study in 47 patients undergoing caval replacement for infiltrating tumor showed that graft replacement of the IVC prevents venous complications in the extremities and probably contributes to improving quality of life and survival [13].

The IVC can also be replaced with autologous veins, but this option is often impractical because of the difficulty in harvesting enough homologous material to substitute a long venous segment. Spiral vein grafts are not recommended owing to the elevated thrombogenicity of the surface exposed at the suture sites and the high risk of veins collapsing due to the positive intra-abdominal pressure [14]. Currently, PTFE grafts are the prosthesis of choice for caval replacement, with ringed grafts best able to prevent side effects during the immediate post-operative period and symptoms of chronic venous insufficiency [10,15].

In the case presented here, caval replacement was carried out in the context of a particular anatomic setting: horseshoe kidney. Central fusion of the kidneys occurs when the embryo is 5 to 12 mm, i.e., when the kidneys lie in the true pelvis. Horseshoe kidney is usually located between L3 and L5, lower than normal, because its ascent is impeded by the root of the inferior mesenteric artery. Also, its normal medial rotation is impeded, frequently resulting in an anomalous course of the ureters, which are often ectopic or retrocaval. The kidneys are fused together by a thick isthmus, generally parenchymatous and very often situated anterior to the aorta and the IVC. More rarely it may be found posterior to these vessels or in the interaortocaval space.

The incidence of horseshoe kidney is between 1/400 and 1/800 persons, with a male-to-female ratio of about 2:1. In 90% of cases, the kidneys are fused at the lower poles, either symmetrically (midline fusion) or asymmetrically (L-shaped fusion) [16].

The vascular network of horseshoe kidney varies widely:

1. One renal artery per side (20%)

2. One renal artery per side with a branch from the aorta to the isthmus (30%)

3. Two arteries per side with an aortic branch to the isthmus (15%)

4. Two arteries per side with one or more arteries from the iliac arteries to the isthmus (15%)

5. Multiple renal arteries originating from the aorta, the iliac and the mesenteric arteries (20%) [17]

Because embryogenesis of the kidney occurs contemporaneously with its venous drainage system in the vena cava, there is a correlation between the presence of horseshoe kidney and venous abnormalities:

1. Double IVC (0.2-3%)

2. Postrenal left IVC (0.2-0.5%)

3. Retroaortic left renal vein (2.1%)

4. Circumaortic left renal vein (8.7%)

5. Retrocaval or circumcaval ureters (0.001-0.1%)

6. Preaortic iliac vein confluence (very rare) [18]

The association between non-seminomatous GCT and horseshoe kidney is a rare event and is seen in about 1.3% of patients requiring retroperitoneal lymph node dissection [16]. To our knowledge, no cases have been reported in which replacement of the IVC was also necessary.

As described by Evans et al. [16] and Sogani et al., [19] retroperitoneal lymph node dissection in patients with horseshoe kidney demands scrupulous preparation because of the added complexities arising from the vascular abnormalities associated with this congenital disorder. Added to this is the need to avoid resecting the isthmus as it is often composed of functional renal parenchyma and is therefore richly vascularized. Only the resection of a fibrous isthmus should not lead to a reduction in renal function or to immediate complications such as bleeding.

Scheduled caval replacement will entail careful preoperative imaging to evaluate the morphology and functionality of the isthmus of horseshoe kidney, its arterial vascularization and anatomic relationships with the ureters and venous structures. In young patients, the surgical goal is to achieve radical removal. Winter et al. [11] reported that, on the basis of univariate analysis, the residual mass size (>5 cm) and the IGCCCG prognostic class were predictive factors for completing residual tumor resection in procedures involving the IVC (Inferior Vena Cava), thus providing the basic parameters for surgical planning. Also of fundamental importance is to minimize major hemodynamic complication and preserve renal function. Residual tumor resection with additional vascular procedures is burdened by high mortality, with survival rates of about 71% due to the deteriorated physical condition of patients who have undergone several cycles of chemotherapy [20,21].

## Conclusions

Residual tumor removal is associated with high rates of mortality and comorbidity. In patients with horseshoe kidney and advanced stage non-seminomatous GCT (NSGCT) and post-chemotherapy retroperitoneal residual disease amenable to resection, the aims of surgical treatment are to achieve radical tumor removal, restore venous drainage, and preserve renal function without worsening the precarious health of a patient already severely debilitated by disease and treatment. These objectives can be reached with scrupulous evaluation of the individual case by a multidisciplinary team composed of an urologist, a vascular surgeon, and a medical oncologist. Caval replacement with a ringed PTFE should be performed without resecting the renal isthmus, owing to the frequent vascular anomalies associated with this disorder, and thus avoid the risk of bleeding complications.

### Consent

Written informed consent was obtained from the patient for the publication of this case presentation and accompanying images. A copy of the written consent is available for review by the Editor-in-Chief of this journal.

## Abbreviations

AFP: Alpha-fetoprotein; BHCG: Beta-human chorionic gonadotropin; GCT: Germ cell tumor; IGCCCG: International Germ Cell Cancer Collaborative Group; IVC: Inferior vena cava; LDH: Lactate dehydrogenase; NSGCT: Non-seminomatous germ cell tumor; PET: Positron-emission tomography; PTFE: Polytetrafluoroethylene.

## Competing interests

The authors declare that they have no competing interests.

## Authors’ contributions

PR, PG and PD wrote the manuscript. PR, DF, PD and PG performed surgery. BL and LC followed clinically the patient in the pre-and post-operative. GV was involved in the final editing. CC and PL still maintain contact with the patient and are aware of information necessary for the review of the work: their contribution to the revision of the work was essential and extremely valuable. All authors approved the final manuscript.

## Pre-publication history

The pre-publication history for this paper can be accessed here:

http://www.biomedcentral.com/1471-2490/14/40/prepub
